# Impact of elexacaftor/tezacaftor/ivacaftor on the small airways in cystic fibrosis

**DOI:** 10.36416/1806-3756/e20240406

**Published:** 2025-05-21

**Authors:** Sofia Campos Silva, Andreia Barroso, Maria Cunha, Elsa Fragoso, Pilar Azevedo

**Affiliations:** 1. Hospital Beatriz Ângelo, ULS Loures-Odivelas, Loures, Portugal.; 2. Hospital de Faro, ULS do Algarve, Faro, Portugal.; 3. Hospital de Vila Franca de Xira, ULS Estuário do Tejo, Vila Franca de Xira, Portugal.; 4. Hospital de Santa Maria, ULS de Santa Maria, Lisboa, Portugal.; 5. Faculdade de Medicina, Universidade de Lisboa, Lisboa, Portugal.

**Keywords:** Cystic fibrosis/diagnostic imaging, Lung/physiopathology, Respiratory function tests, Lung/drug effects, Lung/diagnostic imaging, Cystic fibrosis/drug therapy

## Abstract

**Objective:**

To evaluate the impact of the elexacaftor/tezacaftor/ivacaftor (ELX/TEZ/IVA) combination on the small airways in adults with cystic fibrosis (CF), a genetic disorder that primarily affects the respiratory system, leading to progressive lung disease. In CF, the small airways play a critical role, contributing to chronic symptoms such as cough, sputum production, and dyspnea.

**Methods:**

This was a single-center, retrospective observational study of adults with CF treated with ELX/TEZ/IVA for 12 months. We compared the patients who were homozygous for the F508del mutation of the CF transmembrane conductance regulator (CFTR) gene with those who were heterozygous for that mutation, in terms of lung function outcomes (FEV_1_, FEF_25-75%_, and the RV/TLC ratio) and the extent of non-homogeneous ground-glass opacity. Among the patients within the cohort, the same parameters were evaluated separately in those who had advanced lung disease and in those who had previously undergone CFTR modulator therapy.

**Results:**

There was a significant post-treatment improvement in lung function, with a median increase of 0.42 L/s in the FEF_25-75%_ (p < 0.001) and a 5% reduction in the mean RV/TLC ratio (p < 0.001). There was a trend toward a higher improvement the F508del homozygous patients. A significant reduction in non-homogeneous ground-glass opacity was observed in 79.5% of the patients. Among the patients with advanced lung disease, there were notable post-treatment improvements in all of the parameters assessed.

**Conclusions:**

Our results highlight the positive impact that ELX/TEZ/IVA treatment can have on small airway function in patients with CF, with potential benefits even for those with advanced lung disease. Further research is needed in order to evaluate the long-term effects of this treatment and its relationship with patient-reported outcomes.

## INTRODUCTION

Cystic fibrosis (CF) is a rare genetic disease with autosomal recessive inheritance, caused by variants in the CF transmembrane conductance regulator (*CFTR*) gene, that affects approximately 100,000 people worldwide.^1^ Dysfunction of the CFTR protein leads to the development of multiple manifestations across various organ systems, with the respiratory system being the most prominent. Pulmonary involvement typically causes the most severe, progressive form of CF, ultimately leading to death. Over 2,000 variants have been described, the most common being F508del, which is found in nearly 90% of CF patients, of whom approximately 50% are homozygous for this mutation.^2^

The importance of the small airways in the pathophysiology of lung disease in CF is well known, because CFTR dysfunction causes viscous secretions and failure of mucociliary clearance due to respiratory mucin adherence to the airway epithelium. This leads to the production and accumulation of viscous bronchial secretions, air trapping, and increased respiratory muscle workload. Together, these factors contribute to chronic symptoms such as cough, sputum production, dyspnea, and reduced exercise capacity.^3^

In July of 2021, the CFTR modulator combination elexacaftor/tezacaftor/ivacaftor (ELX/TEZ/IVA) was approved for use in Portugal for patients who are homozygous for the F508del mutation and F508del heterozygous patients carrying a second minimal function mutation; in February of 2022, it was approved for those carrying at least one F508del allele.^4^

The safety and tolerability profile of ELX/TEZ/IVA have been well established, as have its benefits, including improvements in FEV_1_, a reduction in the number of pulmonary exacerbations, and a reduction in the need for inhaled antibiotics,^5^ as well as enhanced quality of life, nutritional status, and survival.^6^ ^,^ ^7^ However, there is still limited knowledge regarding the impact of this combination on the small airways.

In this study, we aimed to identify the functional and imaging outcomes of patients who initiated treatment with the ELX/TEZ/IVA combination, in order to assess its impact on small airways disease. We also aimed to compare the outcomes of ELX/TEZ/IVA treatment between F508del homozygous patients and F508del heterozygous patients.

## METHODS

This was a retrospective, observational, single-center study of adult CF patients followed at the Cystic Fibrosis Referral Center of the Hospital de Santa Maria, in the city of Lisbon, Portugal, who started treatment with ELX/TEZ/IVA and continued it for at least one year. Patient recruitment was conducted between September and November of 2024.

The collected data included age; sex; *CFTR* genotype; date of treatment initiation; prior treatment with other CFTR modulators; lung function, at baseline and after one year of treatment; and imaging findings on chest CT performed prior to the initiation of ELX/TEZ/IVA and after at least one year of treatment. Relevant medical histories were evaluated by reviewing patient medical records.

Lung function parameters were obtained through spirometry and plethysmography. Small airways function was assessed by measuring FEF_25-75%_, expressed in liters/second and as a percentage of the predicted value, calculating the RV/TLC ratio, and looking for a non-homogeneous ground-glass opacity pattern on chest CT. Improvement in the non-homogeneous ground-glass opacity pattern was subjectively assessed by a thoracic radiologist, who evaluated chest CT scans (performed during inspiration and expiration) and compared them to the previous scans. The impact that treatment with ELX/TEZ/IVA had on obstruction of the larger airways was also assessed by measuring FEV_1_, expressed in liters. 

Because the study period overlapped with the transition to new Global Lung Function Initiative equations, some pulmonary function tests (PFTs) were conducted using the old equations and more recent PFTs were conducted using the new equations. Therefore, the analysis of the results focused on the comparison of absolute values, given that the use of different equations for predicting reference values could introduce bias. Advanced lung disease was defined as an FEV_1_ < 40% of the predicted value. 

Lung function data were collected as close as possible to (before) the initiation of ELX/TEZ/IVA therapy (often on the same day). However, due to the constraints imposed by the COVID-19 pandemic, the median time from the pre-treatment lung function evaluation to the initiation of therapy was 82.55 days (IQR: 17.75-102.0 days). For the post-treatment evaluation, the PFT conducted soonest after at least 12 months of therapy was selected. The median time from the initiation of therapy to the follow-up PFT was 439.0 days (IQR: 385.3-491.8 days).

For descriptive statistics, continuous variables with normal distribution are presented as mean ± standard deviation, whereas the remaining data are expressed as median and interquartile range. Categorical variables are reported as absolute and relative frequencies. The mean differences between subgroups were analyzed by t-test for independent samples if the data had a normal distribution and by Mann-Whitney U test if the data had a non-normal distribution. Pre- and post-treatment comparisons of data with normal and non-normal data distribution were made with t-test for paired samples and the Wilcoxon test, respectively. Categorical variables were analyzed by using the chi-square test for independent comparisons and McNemar’s test for paired comparisons. Values of p < 0.05 were considered statistically significant. Statistical analysis was performed with the IBM SPSS Statistics software package, version 28.0 (IBM Corp., Armonk, NY, USA).

## RESULTS

During the study period, a total of 74 adult patients were followed at our referral center for CF. Of those, 55 were eligible for ELX/TEZ/IVA therapy, in agreement with national recommendations. Nine patients were excluded because they had been on the therapy for less than 12 months and 2 because they were on a clinical trial of another CFTR modulator.

**Figure 1:**
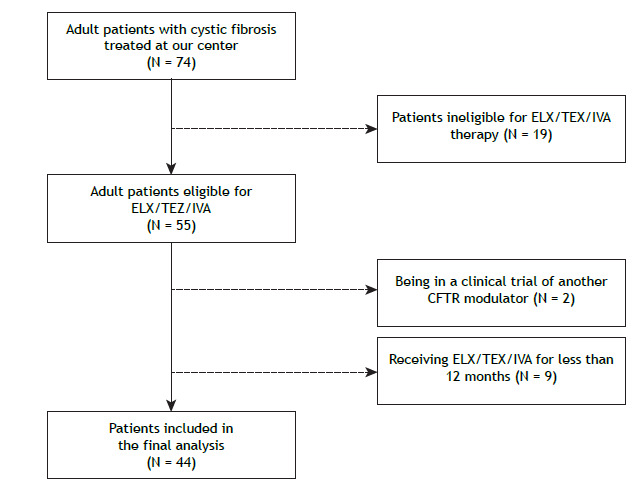
Flow chart of patient recruitment and enrollment. ELX/TEZ/IVA: elexacaftor/tezacaftor/ivacaftor; and CFTR: cystic fibrosis transmembrane conductance regulator.

Patient recruitment, enrollment, and follow-up are depicted in Figure 1. Table 1 summarizes the demographic and clinical features of the study cohort at baseline. The mean age at the diagnosis of CF was 13.4 ± 12.5 years. The mean age at initiation of ELX/TEZ/IVA therapy was 31.9 ± 12.5 years. Advanced lung disease was seen in 8 (18.2%) of the 44 patients.

**Table 1:** Clinical and demographic characteristics of the patients included in the study.

Variable	(N = 44)
Sex, n (%)	
Female	22 (50.0)
Male	22 (50.0)
Age (years) at CF diagnosis, mean ± SD	13.4 ± 12.5
Age (years) at ELX/TEZ/IVA initiation, mean ± SD	31.9 ± 12.5
*CFTR* genotype, n (%)	
Homozygous F508del	17 (38.6)
F508del/R334W	7 (15.9)
F508del/P205S	4 (9.1)
F508del/R1066C	2 (4.5)
Other heterozygous F508del	14 (31.8)
Prior CFTR modulator therapy^a^, n (%)	11 (25.0)
Advanced lung disease, n (%)	8 (18.2)

ELX/TEZ/IVA: elexacaftor/tezacaftor/ivacaftor; and CFTR: cystic fibrosis transmembrane conductance regulator. ^a^Two patients were previously treated with TEZ/IVA, and nine were previously treated with lumacaftor/IVA.

After 12 months of ELX/TEZ/IVA therapy, there was a median increase of 0.42 L/s in the FEF_25-75%_ (p < 0.001) and a 5% reduction in the mean RV/TLC ratio (p < 0.001). A post-treatment improvement in the non-homogeneous ground-glass opacity pattern was observed in 35 (79.5%) of the 44 patients evaluated. The median time from the initiation of therapy to the follow-up chest CT was 623.0 days (IQR: 424.3-795.0 days). Of the 17 patients in the F508del homozygous group, 14 (82.4%) showed improvement in that pattern, compared with 77.8% of the 27 patients in the heterozygous group. Even among the 8 patients with advanced lung disease, 7 (87.5%) exhibited radiological improvement in the pattern. Among the 11 patients previously treated with other CFTR modulators, 8 (72.7%) showed radiological improvement in the non-homogeneous ground-glass opacity pattern.

**Figure 2:**
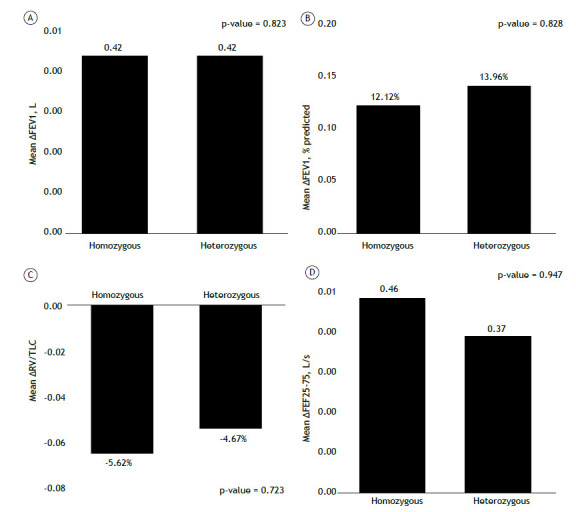
Differences in outcomes between F508del homozygous and F508del heterozygous patients.

The degree of improvement in FEV_1_, as an absolute value and as a percentage of the predicted value, was similar between the F508del homozygous patients and the F508del heterozygous patients (Table 2). As shown in Figure 2, the improvements in the small airways, as assessed by measuring the FEF_25-75%_ (in L/s) and the RV/TLC ratio, were greater in the F508del homozygous patients, although the differences in comparison with the F508del heterozygous patients were not statistically significant (p = 0.947 and p = 0.723, respectively).

**Table 2:** Effects of elexacaftor/tezacaftor/ivacaftor treatment on FEV_1_, FEF_25-75%_, and the RV/TLC ratio in patients with cystic fibrosis (N = 44).

Group	Outcome measure	Baseline	Follow-up	Difference	p
		Value [95% CI]	Value [95% CI]	Value [95% CI]	
Overall	FEV_1_ (L), mean	2.06 [1.80 to 2.33]	2.49 [2.18 to 2.76]	0.42 [0.28 to 0.57]	< 0.001
	FEV_1_ (% pred.), mean	60.11 [53.92 to 66.30]	73.36 [66.46 to 80.26]	13.25 [9.25 to 17.25]	< 0.001
	FEF_25-75%_ (L/s), median	0.98 [0.91 to 1.42]	1.22 [1.33 to 2.11]	0.42 [0.22 to 0.70]	< 0.001
	FEF_25-75%_ (% pred.), median	24.00 [21.88 to 34.51]	31.00 [32.22 to 49.33]	10.0 [6.50 to 15.50]	< 0.001
	RV/TLC ratio, mean	44.80 [41.06 to 49.29]	39.80 [35.53 to 42.31]	−5.0 [−7.55 to −2.44]	< 0.001
F508del homozygous	FEV_1_ (L), mean	2.19 [1.57 to 2.54]	2.61 [1.94 to 2.92]	0.42 [0.22 to 0.63]	0.01
	FEV_1_ (% pred.), mean	64.76 [48.73 to 73.74]	76.88 [61.22 to 84.01]	12.12 [6.90 to 17.40]	< 0.001
	FEF_25-75%_ (L/s), median	1.42 [0.98 to 1.83]	1.45 [1.33 to 2.47]	0.46 [0.10 to 0.81]	0.010
	FEF_25-75%_ (% pred.), median	35.50 [22.92 to 45.58]	39.00 [31.49 to 58.15]	10.00 [0.30 to 17.00]	0.009
	RV/TLC ratio, mean	43.37 [36.82 to 49.85]	37.72 [31.09 to 44.35]	−5.62 [−8.66 to −2.58]	0.002
F508del heterozygous	FEV_1_ (L), mean	1.99 [1.60 to 2.37]	2.41 [1.97 to 2.87]	0.42 [0.22 to 0.63]	< 0.001
	FEV_1_ (% pred.), mean	57.19 [49.51 to 67.24]	71.15 [62.03 to 83.30]	13.96 [8.09 to 19.84]	< 0.001
	FEF_25-75%_ (L/s), median	0.73 [0.68 to 1.34]	1.11 [1.06 to 2.15]	0.37 [0.13 to 1.25]	0.001
	FEF_25-75%_ (% pred.), median	18.60 [16.65 to 31.91]	27.00 [26.49 to 49.95]	10.00 [5.5 to 20.5]	< 0.001
	RV/TLC ratio, mean	45.60 [39.92 to 51.27]	40.93 [36.38 to 45.48]	−4.67 [−8.40 to −0.93]	0.017
Advanced lung disease	FEV_1_ (L), mean	1.00 [0.82 to 1.10]	1.34 [1.03 to 1.51]	0.34 [0.17 to 0.52]	0.019
	FEV_1_ (% pred.), mean	33.38 [28.76 to 36.37]	45.63 [34.27 to 53.73]	12.25 [5.49 to 19.01]	0.036
	FEF_25-75%_ (L/s), median	0.43 [0.31 to 0.59]	0.54 [0.38 to 0.90]	0.16 [0.02 to 0.36]	0.028
	FEF_25-75%_ (% pred.), median	10.50 [9.0 to 14.00]	14.00 [11.00 to 23.00]	4.50 [5.00 to 9.00]	0.034
	RV/TLC ratio, mean	62.23 [55.05 to 69.42]	54.73 [45.10 to 64.36]	−7.51 [−11.37 to −3.64]	0.002
Prior CFTR modulator therapy	FEV_1_ (L), mean	2.36 [1.55 to 2.88]	2.71 [1.76 to 3.08]	0.35 [0.08 to 0.61]	0.016
	FEV_1_ (% pred.), mean	71.36 [51.98 to 85.17]	81.27 [59.10 to 92.62]	9.91 [3.02 to 16.80]	0.009
	FEF_25-75%_ (L/s), median	1.35 [1.14 to 3.12]	1.46 [1.07 to 2.31]	0.38 [0.14 to 0.82]	0.139
	FEF_25-75%_ (% pred.), median	37.00 [27.00 to 49.96]	39.00 [29.00 to 64.50]	8.00 [2.0 to 17.0]	0.109
	RV/TLC ratio, mean	39.26 [33.26 to 45.25]	34.51 [29.39 to 39.64]	−4.74 [−10.17 to −0.68]	0.076

% pred.: percentage of the predicted value.

## DISCUSSION

The benefits of highly effective CFTR modulator therapy for lung function, particularly for improving FEV_1_, are well-established in the literature.^7^ ^-^ ^10^ To our knowledge, data on the impact of such therapies on the small airways are sparse. In this real-world study, we have provided clear evidence of the positive impact that 12 months of treatment with ELX/TEZ/IVA has on the small airways, observing a median improvement of 0.42 L/s in the FEF_25-75%_ and a 5% reduction in the mean RV/TLC ratio. When evaluating the impact on patients with advanced lung disease, we observed statistically significant improvements across all parameters assessed. The greatest improvement was seen in the RV/TLC ratio, whereas the improvement in the FEF_25-75%_, albeit statistically significant, was more modest.

Although trends in improvement in the small airways were observed in patients previously treated with other CFTR modulators, those changes were not statistically significant. That might reflect a gain achieved with previous treatment, rendering further improvement more challenging to assess.

We also observed improvements in the non-homogeneous ground-glass opacity pattern on chest CT, in homozygous and heterozygous patients, as well as in those previously treated with other CFTR modulators, and even in patients with advanced lung disease.

These results highlight the potential of ELX/TEZ/IVA therapy to target small airway dysfunction, which is critical in the management of CF. Further studies are needed in order to assess the long-term effects of this therapy on the small airways, as well as its relationship with patient-reported outcomes.
